# The Evolution of Flavonoid Biosynthesis: A Bryophyte Perspective

**DOI:** 10.3389/fpls.2020.00007

**Published:** 2020-02-04

**Authors:** Kevin M. Davies, Rubina Jibran, Yanfei Zhou, Nick W. Albert, David A. Brummell, Brian R. Jordan, John L. Bowman, Kathy E. Schwinn

**Affiliations:** ^1^ The New Zealand Institute for Plant and Food Research Limited, Palmerston North, New Zealand; ^2^ Faculty of Agriculture and Life Sciences, Lincoln University, Christchurch, New Zealand; ^3^ School of Biological Sciences, Monash University, Melbourne, VIC, Australia

**Keywords:** anthocyanin, auronidin, dirigent, polyphenol oxidase, transcription factor

## Abstract

The flavonoid pathway is one of the best characterized specialized metabolite pathways of plants. In angiosperms, the flavonoids have varied roles in assisting with tolerance to abiotic stress and are also key for signaling to pollinators and seed dispersal agents. The pathway is thought to be specific to land plants and to have arisen during the period of land colonization around 550–470 million years ago. In this review we consider current knowledge of the flavonoid pathway in the bryophytes, consisting of the liverworts, hornworts, and mosses. The pathway is less characterized for bryophytes than angiosperms, and the first genetic and molecular studies on bryophytes are finding both commonalities and significant differences in flavonoid biosynthesis and pathway regulation between angiosperms and bryophytes. This includes biosynthetic pathway branches specific to each plant group and the apparent complete absence of flavonoids from the hornworts.

## Opening Comments

**Figure 1:**
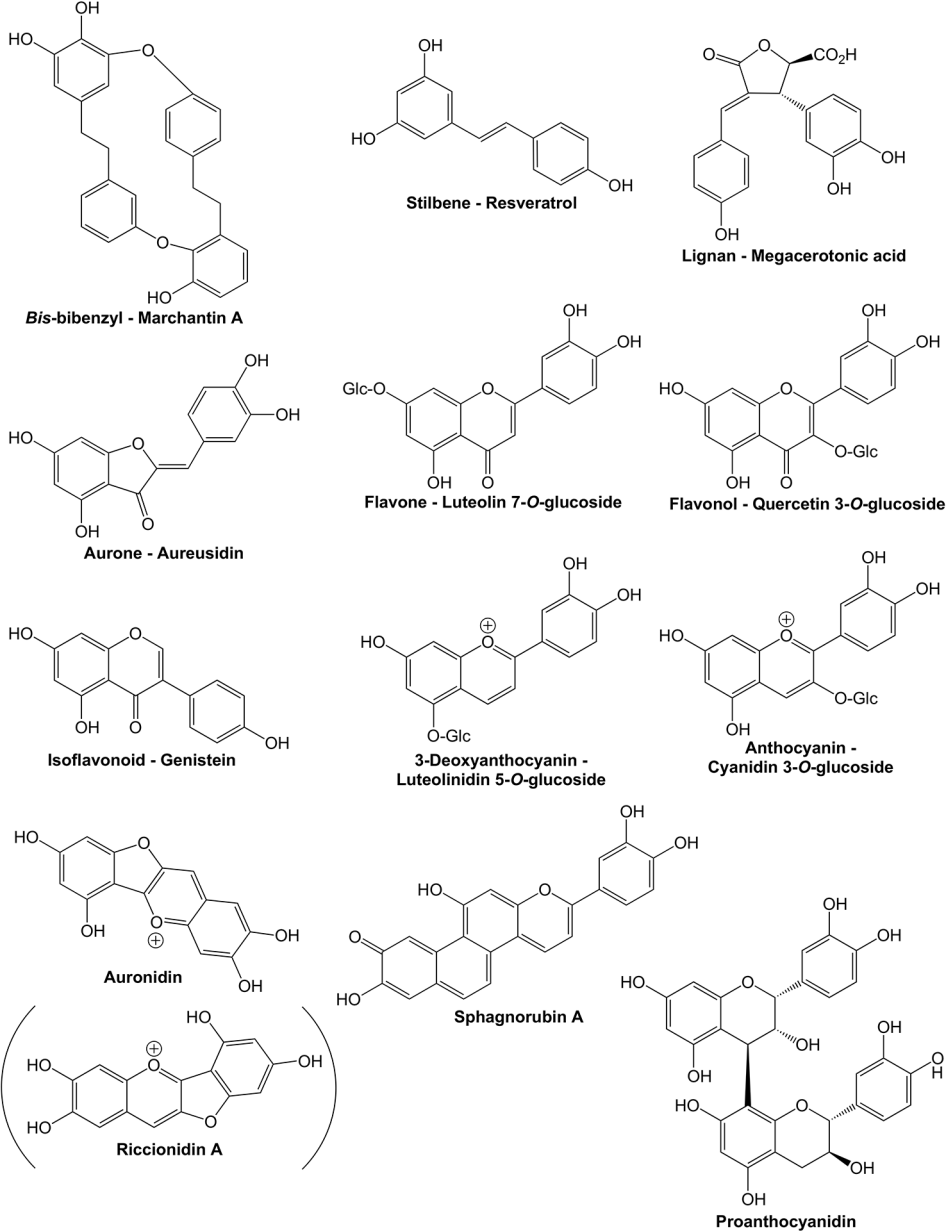
Examples of compounds from phenylpropanoid pathway branches discussed in the text. Bibenzyls, stilbenes, and lignans are phenylpropanoids formed from branches prior to the start of the specific flavonoid pathway branch. The example compounds are from liverworts, angiosperms, and hornworts, respectively. All the other compounds shown are flavonoids.

**Figure 2:**
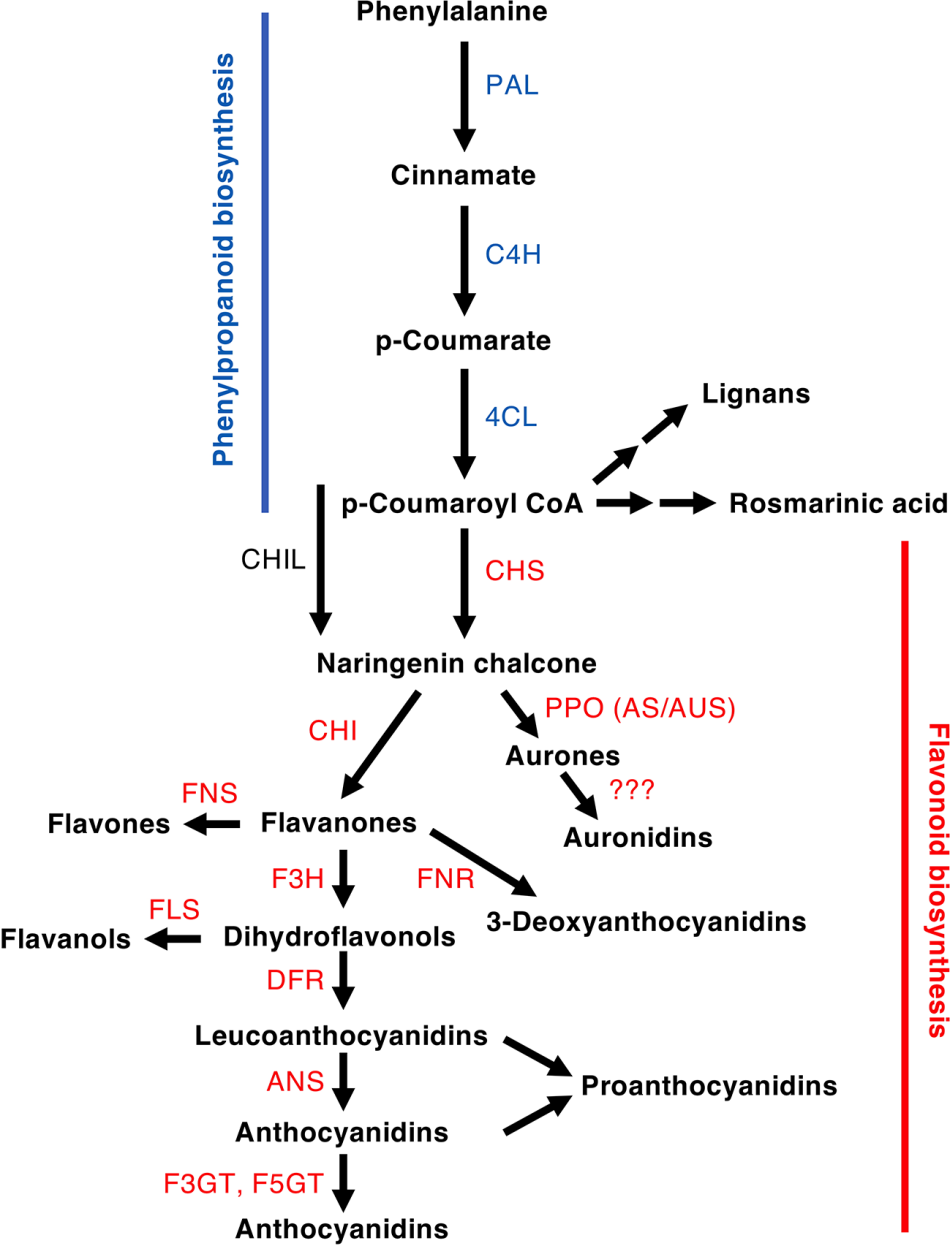
Schematic of a section of the phenylpropanoid pathway leading to the production of major compound groups discussed in the article. Enzyme abbreviations are: PAL, phenylalanine ammonia-lyase; C4H, cinnamate 4-hydroxylase; 4CL, 4-coumarate:CoA ligase; CHS, chalcone synthase; CHI, chalcone isomerase; CHIL, chalcone isomerase-like; F3H, flavanone 3-hydroxylase; DFR, dihydroflavonol 4-reductase; FNR, flavanone reductase; ANS, anthocyanidin synthase; F3GT, flavonoid 3-*O*-glucosyltransferase; A5GT, anthocyanidin 5-*O*-lucosyltransferase; AUS/AS, aureusidin/aurone synthase; FNS, flavone synthase (2OGD = I, Cyp450 = II); FLS, flavonol synthase.

The flavonoid pathway is a core component enabling land plants to interact with their environment. Flavonoids have been demonstrated to assist in tolerance to both abiotic and biotic stresses, and their production can be induced by cold, UV-B light (UVB) or strong white light, nutrient deprivation, desiccation, salinity, metal toxicity, and pest and pathogen attack (Agati and Tattini, 2010; Cheynier et al., 2013; Landi et al., 2015; Davies et al., 2018). In angiosperms, flavonoids are also key for signaling to pollinators and seed dispersal agents. Flavonoids are formed within the larger phenylpropanoid pathway, starting with chalcones as the first flavonoids. During plant evolution, flavonoid pathway diversity has increased greatly, with more than 8,000 different structures reported from the relatively small number of plant species studied to date (Andersen and Markham, 2006). This multiplicity of structures and functions is thought to have assisted land plants to colonize the wide range of environments they now occupy. The over 8,000 compounds are grouped into relatively few classes of flavonoids, based on the core structure and/or biosynthetic origin. The major flavonoid classes are the flavones, flavonols, isoflavonoids, aurones, 3-deoxyanthocyanins, anthocyanins, proanthocyanidins (condensed tannins), and the recently reported “auronidins” (Berland et al., 2019) (**Figures 1** and **2**). However, there are also notable groups of related non-flavonoid compounds produced with the same starting precursors as used for chalcone formation, such as the stilbenes and bibenzyls. Most flavonoids are targeted to the vacuole as water-soluble glycosides, although some are transported to the cell wall or are released from the plant to the environment. Many can absorb light in the UV-spectrum, while anthocyanins and auronidins provide colored pigments that can screen in the visible part of the light spectrum (Lee and Gould, 2002; Landi et al., 2015; Berland et al., 2019).

Flavonoid biosynthesis is frequently considered unique to land plants. Some charophycean algae do tolerate terrestrial conditions (Karsten and Holzinger, 2014; Holzinger and Pichrtová, 2016) and can produce polyphenolics in response to abiotic stresses such as UVB, salinity, or dehydration. Indeed, one hypothesis is that land plants arose from algal ancestors that were already terrestrial (Harholt et al., 2016) and therefore may have had some of the biosynthetic pathways characteristic of land plants. However, there are as yet no convincing reports of the flavonoid pathway branch existing outside of land plants, with the possible exception of some fungi. A common adaptation for UVB tolerance in extant algae is the production of mycosporine-like amino acids (MAA). However, while MAA production is frequently reported for red algae (Rhodophyta) and other marine organisms, there are few reports for the Chlorophyta (a green algal clade that with Streptophyta form the Viridiplantae) and none for the charophyte algae that is thought to be most closely related to the land plant ancestor. Although genes for the initial steps of the phenylpropanoid pathway may be present in the genomes of extant Rhodophyta, Glaucophyta, Chlorophyta, and charophytes (Labeeuw et al., 2015; de Vries et al., 2017; Davies et al., 2020), there are no substantiated examples of flavonoid-specific genes being identified. Indeed, many metabolite studies on algae report only amounts of “total flavonoids” measured using general assays. Nevertheless, there are some reports detailing specific flavonoid structures in algal preparations, including compounds such as chalcones, flavones, flavonols, isoflavonoids, and proanthocyanidins (Klejdus et al., 2010; Goiris et al., 2014; El Shoubaky et al., 2016; Agregán et al., 2017; Ben Saad et al., 2017). Although the concentrations of flavonoid compounds reported in most examples are extremely low compared with those commonly found in land plants (ng gDW^−1^ amounts compared with mg gDW^−1^), these reports mean that the presence of a biosynthetic pathway to flavonoids in algae cannot be ruled out.

The phenolics most commonly produced by algae are the phlorotannins, diverse oligomers derived from phloroglucinol that are found in brown algae (Heterokonts) (Imbs and Zvyagintseva, 2018). In *Ectocarpus siliculosus*, the phloroglucinol precursor is formed by the condensation of malonyl-CoA by a polyketide synthase (PKS) (Meslet-Cladière et al., 2013). This is analogous to the action of CHALCONE SYNTHASE (CHS) in the condensation of malonyl-CoA with a *p*-coumaroyl-CoA starter molecule for flavonoid biosynthesis. However, given the very large phylogenetic distance between brown algae and land plants this may be an example of parallel evolution. Notably, in fungal species there have been recent reports of both the production of flavonoids and the presence of genes with significant sequence similarity to those of the phenylpropanoid pathway of land plants. Findings include the detection of a range of phenylpropanoids, including flavonoids, in *Fusarium* (Bilska et al., 2018) and detection of flavonoids and candidate genes for stilbene production in *Alternaria* (Lu et al., 2019). The significance of phenylpropanoid biosynthesis being present in fungi for the current proposals on the evolutionary origins of flavonoid biosynthesis has yet to be addressed. The separation of the fungi and the algae/plant ancestors is thought to be an ancient event, preceding the divergence fungi and animals (Burki, 2014; Burki et al., 2020).

### Origins and Vegetative Functions of the Flavonoid Pathway

Regardless of whether the ancestral genes for flavonoid biosynthesis were present in algal ancestors, the flavonoid pathway we see in extant land plants is hypothesized to have arisen when the land plant ancestors were first colonizing the land about 550–470 million years ago (MYA) (Markham, 1988; Stafford, 1991; Jorgensen, 1994; Koes et al., 1994; Kenrick and Crane, 1997; Rozema et al., 2002). Two major hypotheses have been proposed for the initial role of flavonoids. Firstly, that flavonoids may have helped in coping with the additional abiotic stresses resulting from a terrestrial lifestyle, in particular increased exposure to UVB, but potentially also drought and extreme temperature fluctuations (Markham, 1988; Jorgensen, 1994; Kenrick and Crane, 1997; Cockell and Knowland, 1999; Rozema et al., 2002; Ligrone et al., 2012; Mouradov and Spangenberg, 2014; Demarsy et al., 2017; Davies et al., 2018; de Vries and Archibald, 2018; Rensing, 2018). The alternative proposal is that flavonoids arose as physiological regulators or chemical messengers. This was outlined in Stafford (1991) for the regulation of auxin action, with signaling to mycorrhizal and symbiotic fungi proposed as possible additional communication functions. It was argued that flavonoids would probably have been present at only low concentrations when the pathway first evolved, limiting their efficacy as UVB-screening compounds. This was in the context of other arguments against the need for flavonoids as UVB-screens, such as the effective UVB-screening properties of non-flavonoid phenylpropanoids like the hydroxycinnamic acids (HCAs). More recently, arguments for the early functions of flavonoids being other than UVB-screening have been extended by discoveries on their antioxidant properties and possible signaling actions through the redox pathway or by affecting H_2_O_2_ retrograde signals between the chloroplast and nucleus (Taylor and Grotewold, 2005; Agati and Tattini, 2010; Pollastri and Tattini, 2011; Agati et al., 2012; Brunetti et al., 2018; Foyer, 2018; Muhlemann et al., 2018; Brunetti et al., 2019).

That flavonoids affect auxin transport has now been demonstrated in several angiosperm species, including Arabidopsis, apple, and tomato, by analysis of mutants or transgenic lines with reduced flavonoid biosynthesis (Brown et al., 2001; Buer and Muday, 2004; Taylor and Grotewold, 2005; Peer and Murphy, 2007; Dare and Hellens, 2013; Maloney et al., 2014). Altered developmental traits in such plants include dwarfing, loss of pollen fertility and altered root development and gravitropic responses (Van der Meer et al., 1992; Napoli et al., 1999; Brown et al., 2001; Dare and Hellens, 2013; Maloney et al., 2014; Muhlemann et al., 2018). The phenotypes observed vary between species, for example the complete loss of flavonoids in the Arabidopsis *chs* mutant affects root patterning but not pollen viability (Burbulis et al., 1996; Ylstra et al., 1996). To date, the great majority of data are for angiosperms, and studies on other plant groups are required to determine whether these flavonoid functions are shared across land plants and so may have a common evolutionary origin in the early land plant ancestor. Differential distribution of flavonols and auxin has been observed accompanying stem reorientation in a gymnosperm (Ramos et al., 2016), supporting a conserved function in auxin transport within seed plants. If the genetic mechanisms involved are also conserved, then that would support an evolutionary origin before 350–300 MYA. However, while definitive experiments on flavonoids and hormone function have not been conducted in bryophytes, indications are that flavonoids are not necessary for normal development in this plant group. No flavonoids have been detected to date in hornworts, and a genetic mutant of the liverwort *Marchantia polymorph*a lacking flavonoids has normal developmental patterns (Clayton et al., 2018). Addition of phenylpropanoids or phenylpropanoid pathway inhibitors can alter bryophyte development in culture, but whether this is because of altered hormone action has not been tested (Chattopadhyay et al., 2018). Even with additional data it may be difficult to determine the most probable option between developmental roles for flavonoids having been acquired in seed plants since the last common ancestor, or having being present but then lost during subsequent evolution of the bryophytes. Nevertheless, establishing whether flavonoids regulate auxin action in bryophytes is an important goal.

**Figure 3:**
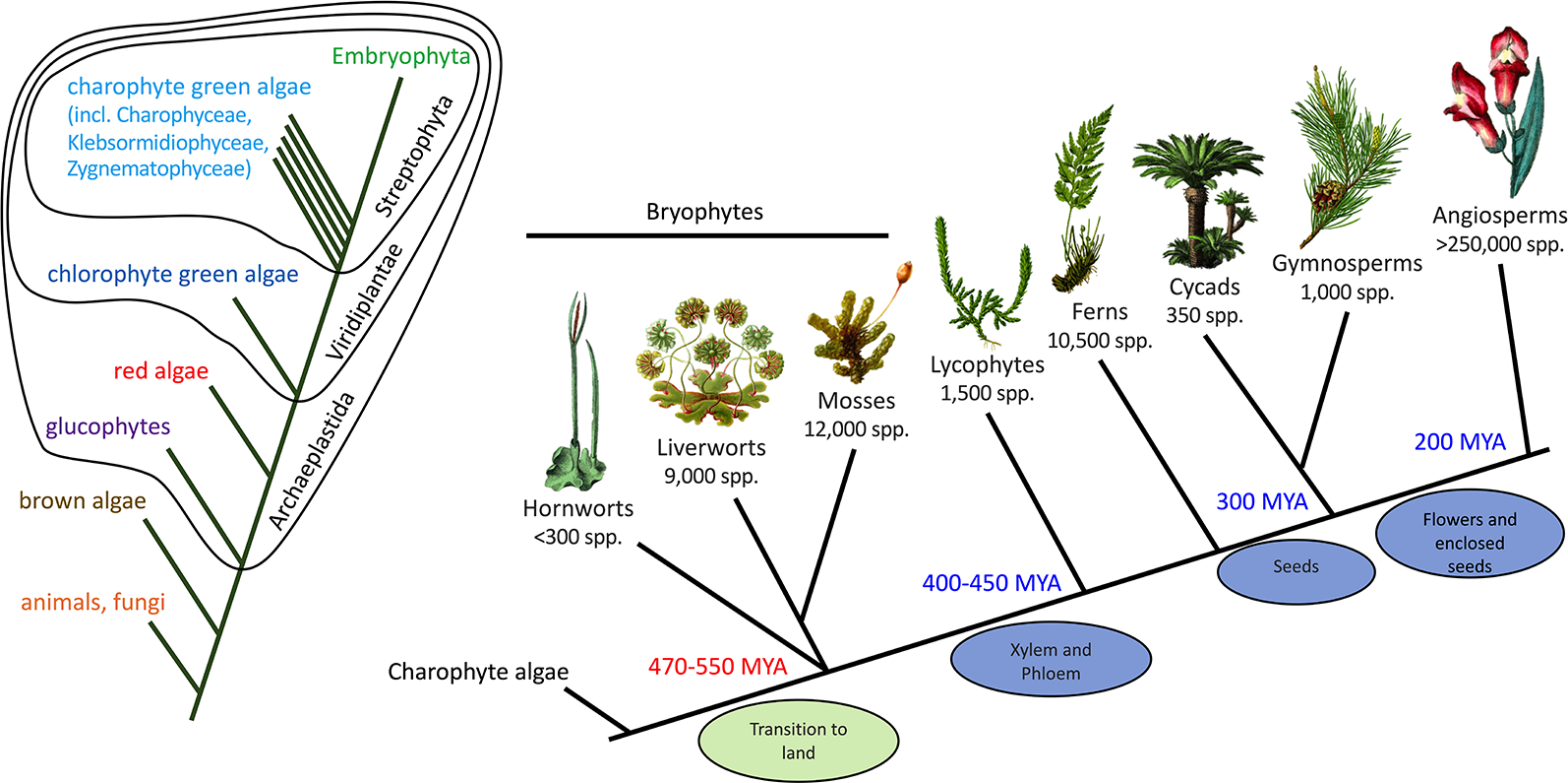
Phylogenetic context of land plants. The phylogenetic tree on the left shows the relationships of the animals, fungi, and plants, including the major divisions within the plants. The tree on the right shows the relationships among the land plants (Embryophyta). The phylogeny of the bryophytes is unresolved, but the current proposal of a sister relationship between liverworts and mosses is shown. MYA, million years ago.

Fossils, such as those found in the Rhynie Chert in Scotland (about 410 MYA) (Ligrone et al., 2012), provide detail of the structure of early land plants but little information on their specialized/secondary metabolism. The presence of specialized biosynthetic or storage structures in fossils, such as possible equivalents to the terpenoid-accumulating oil bodies of extant liverworts (Labandeira and Currano, 2013), can support the presence of specialized metabolite pathways but not provide details of the specific compounds produced. To generate hypotheses on the origins and subsequent evolution of the flavonoid pathway we need to compare the genetics and biochemistry of the pathway across diverse extant plant groups, as this can identify conserved pathway components that may have originated with the last common ancestor. In this respect, bryophytes are of key importance (**Figure 3**). “Bryophytes” is the collective name for non-vascular land plants, comprising the liverworts (Marchantiophyta, approximately 9,000 species), hornworts (Anthocerotophyta, approximately 300 species) and mosses (Bryophyta, approximately 12,000 species). Evidence such as morphological comparison with the fossil record has placed liverworts as the “sister” group to extant land plants—that is, at the base of the land plant evolutionary tree, making bryophytes paraphyletic. However, DNA sequencing data have suggested alternatives, in particular, either paraphyletic bryophytes with hornworts as a sister group to all other land plants, or a single monophyletic bryophyte clade that is sister to the vascular plants (Wickett et al., 2014; Puttick et al., 2018). It is generally accepted that land plants evolved from an ancestral charophyte, and the extant algal sister group is probably the order Zygnematales or a clade of the Zygnematales and Coleochaetales together (Zhong et al., 2014; Delwiche and Cooper, 2015; de Vries and Archibald, 2018).

### Flavonoids and Tolerance to Ultraviolet B Light

The origin of the flavonoid pathway for providing tolerance to UVB has been supported by recent studies on the liverwort species Marchantia. (“Marchantia” is used in this article to refer to *M. polymorpha* subsp. *ruderalis*, which is the model experimental species.) Marchantia is an excellent research model: it is small; has a rapid growth rate; can asexually reproduce in large numbers through single-cell-derived clonal gemmae; and, has a small genome (approximately 220 Mb) which, although larger than that of Arabidopsis (at 135 Mb), contains significantly fewer genes (around 19,000 gene models compared with around 28,000 protein-coding genes in Arabidopsis) (Ishizaki et al., 2015; Bowman et al., 2016; Shimamura, 2016; Bowman et al., 2017). It also offers efficient CRISPR/Cas9 mutagenesis in a dominant haploid gametophytic generation (Sugano et al., 2018).

Significantly, the UVB response of Marchantia has many components in common with that of Arabidopsis. Flavonol *O*-glycosides are key for UVB tolerance of Arabidopsis (Kusano et al., 2011; Morales et al., 2013; Yin and Ulm, 2017), while the related flavone *O*-glycosides contribute to Marchantia UVB tolerance (Clayton et al., 2018). In both Arabidopsis and Marchantia, mutants with reduced flavonoid production are more easily damaged by UVB, while mutants or transgenics with increased flavonoid content have increased UVB tolerance. Moreover, the signaling pathway for flavonoid pathway induction through the UV RESISTANCE LOCUS8 (UVR8) photoreceptor, the bZIP transcription factor (TF) ELONGATED HYPOCOTYL5 (HY5), and the modifiers of protein stability such as CONSTITUTIVELY PHOTOMORPHOGENIC1 (COP1) and REPRESSOR OF UVB PHOTOMORPHOGENESIS1 (RUP1) is also conserved between the species (Clayton et al., 2018; Kondou et al., 2019). Equivalent functional studies are lacking on other major basal plant groups, such as mosses and lycophytes. However, phenolics do seem to be important for UVB tolerance in mosses (Clarke and Robinson, 2008; Wolf et al., 2010; Waterman et al., 2017; Soriano et al., 2018b); and UVB exposure of the Antarctic moss *Pohlia nutans* increased transcript abundance for genes of the UVR8 and flavonoid pathways (Li et al., 2019). This suggests that the core UVB protection mechanism of UVR8-induced flavonoid production may already have been established in the last common ancestor of bryophytes and angiosperms. While some of the same genetic components, such as *UVR8* and *HY5*-like genes have been identified in distantly related groups within the Viridiplantae (Allorent et al., 2016; Bowman et al., 2017; Clayton et al., 2018; Soriano et al., 2018a; Kondou et al., 2019), and algae can produce purple phenolic pigments in response to abiotic stress (Aigner et al., 2013; Holzinger and Pichrtová, 2016), UVB-induced flavonoid production has not been characterized in algae. Thus, the UVR8-induction pathway for UVB-absorbing flavonoids (potentially flavone glycosides as the first compounds) may have been a character rapidly acquired during the water-to-land transition.

There are variations among land plants to the UVR8/flavonoid system for providing tolerance to UVB exposure, and further research would be beneficial to establish whether these may also have an early evolutionary origin. In *Arabidopsis*, the HCA compounds sinapate esters have an important role in UVB-screening. Comparison of *Arabidopsis* mutants at different biosynthetic steps found sinapate esters have a protective role comparable to, or perhaps more important than, that of flavonols (Li et al., 1993; Landry et al., 1995). Furthermore, a genetic screen for UVB-tolerance genes identified the transcriptional repressor At*MYB4* as being downregulated in response to UVB to facilitate increased sinapate ester production (Jin et al., 2000). The principal absorption maxima from 290 to 330 nm makes HCAs particularly effective UVB screening compounds, and would be a more carbon-efficient screen than the flavonoids. In addition to flavonol glycosides, mosses have been shown to produce biflavonoids or cell wall-bound phenolics in response to UVB exposure (Clarke and Robinson, 2008; Wolf et al., 2010; Waterman et al., 2017; Soriano et al., 2018b). Biflavonoid induction is a notable feature of the moss *Ceratodon purpureus*, a species found from Antarctica to hot desert environments (Waterman et al., 2017). The production of cell wall-localized phenolics as part of the UVB screening capacity may be a common feature of bryophytes, including for the flavonoid-lacking hornworts (Monforte et al., 2018; Soriano et al., 2018c).

### Pigmented Flavonoids and Tolerance to Abiotic Stress

The other major group of flavonoids shown to be involved in tolerance to abiotic stress is the 3-hydroxyanthocyanins (typically cyanidin-derivatives) that are found in gymnosperms and almost all angiosperms (Lee and Gould, 2002; Landi et al., 2015). Under the physiological conditions commonly found in vegetative tissues, these provide red pigmentation. The structurally similar 3-deoxyanthocyanins have been extensively characterized in ferns, and also reported for lycophytes and mosses (Andersen and Markham, 2006). Although the color of 3-deoxyanthocyanins is shifted toward orange compared to the equivalent 3-hydroxyanthocyanins, in vegetative tissues similar red pigmentation typically occurs from both compound types. Additionally, cell wall-bound red flavonoids have been reported from liverworts and mosses: riccionidin (an auronidin) and sphagnorubin, respectively (Vowinkel, 1975; Kunz et al., 1993; Berland et al., 2019). Until recently, riccionidin and sphagnorubin were considered anthocyanidins (the non-glycosylated anthocyanin core molecule) with additional rings. Thus, an evolutionary path could be envisioned of cell-wall bound anthocyanidin being the basal state that could have been present in the last common ancestor, then a progression to 3-deoxyanthocyanins, and then 3-hydroxyanthocyanins. However, now we know that riccionidins represent a separate flavonoid class unrelated to anthocyanins (see later sections for biosynthetic details) it is less clear as to what the class of red flavonoid pigment (if any) was present in the early land plants. Auronidin and anthocyanin biosynthesis may be derived characters in each lineage, or the missing pathway in each linage may have been lost during evolution. Furthermore, no proanthocyanidins have been reported from bryophytes or lycophytes, suggesting this branch of flavonoid biosynthesis probably arose later in vascular plant evolution, since proanthocyanidins are found in extant ferns, gymnosperms, and angiosperms.

With regard to function, given the lack of flowers or enclosed seeds in the early land plants, any initial functions of pigmented flavonoids were probably unrelated to animal interactions. In vascular plants anthocyanins have been linked to improving tolerance to a range of abiotic stresses, as well as some biotic challenges. However, how anthocyanins achieve this in the different stress situations, and whether there is a single mechanism or specific functional variations, is the subject of much research and debate. Anthocyanins, auronidins and sphagnorubins can screen out white light to reduce photooxidative damage, and it has been estimated that anthocyanins could absorb over 40% of photosynthetically active radiation in the range containing the most damaging wavelengths for photoinhibition (Merzlyak and Chivkunova, 2000; Pietrini et al., 2002). However, anthocyanins can simultaneously reduce the cellular stress associated with photooxidation through quenching reactive oxygen species (ROS). The relative importance of these two mechanisms is unresolved, even for extensively studied situations such as the appearance of red anthocyanins during autumn senescence of leaves of deciduous trees. Localization of the pigmented flavonoids in the cell wall and vacuole could be thought to argue for a screening mechanism, as ROS are principally generated in plastids and mitochondria. However, it has been suggested that this does not rule out an antioxidant primary function (Agati et al., 2012). Many of the same arguments for or against light screening *versus* antioxidant primary functions can also be applied to the function of flavones/flavonols in UVB tolerance (Agati and Tattini, 2010; Agati et al., 2012; Davies et al., 2018). Further complicating the development of a unifying theory for anthocyanin abiotic stress function are alternative hypotheses that involve neither light screening nor ROS scavenging, such as drought tolerance through decreased osmotic potential, increasing light absorption to help warm leaves, providing camouflage against insect herbivores, “honest” signaling to herbivores that leaves contain anti-feedant compounds and/or are about to be shed, and making leaves more noticeable to insect predators (anti-crypsis) (Gould et al., 1995; Lee and Gould, 2002; Manetas, 2006; Archetti, 2009; Hughes, 2011; Agati et al., 2012; Landi et al., 2015; Davies et al., 2018). Additionally, as mentioned earlier, there is also evidence supporting flavonoid roles as signaling molecules (Taylor and Grotewold, 2005; Agati and Tattini, 2010; Agati et al., 2012; Foyer, 2018).

The cell wall-bound nature of auronidins and sphagnorubins complicates the theory of an antioxidant or signaling role for red flavonoids. The cell wall-localization of the red pigments of mosses and liverworts, termed “tissue fixed”, has been known from early studies on *Sphagnum* and various liverworts (Nagai, 1915; Rudolph et al., 1977). Nevertheless, that the environmental stimuli inducing cell wall-bound red pigments in liverworts and mosses appear similar to those that trigger anthocyanin production in vascular plants, was also noted as far back as 1915 (Nagai, 1915). Kny (1890) and Cesares Gil (1902) noted that *M. polymorpha* or *Reboulia hemisphaerica*, respectively, grown in sunny locations produced more pigment than those growing in shady environments. Nagai (1915) was able to show that limiting nitrogen and phosphorus supply intensified the pigmentation of *M. polymorpha* and *M. paleacea*, but upon transfer to nutrient-rich media the newly developed tissue lacked pigmentation. Moreover, combinations of stresses that can cause oxidative stress are strong signals for red flavonoid biosynthesis in many bryophytes. Thus, as with angiosperms, in liverworts and mosses cold and light can individually induce reddening, but strong sunlight in cold conditions induces much stronger pigmentation, whether this be at altitude, in the Antarctic, or during the cold nights and bright days of autumn (Gerdol, 1996; Gerdol et al., 1998; Newsham et al., 2005; Hooijmaijers and Gould, 2007; Glime, 2007; Bonnett et al., 2010).

Detailed studies on Marchantia (Albert et al., 2018; Kubo et al., 2018) and *Ricciocarpos natans* (Kunz and Becker, 1995) found nitrogen deprivation and increased white light exposure induced auronidin accumulation, as has also been shown for anthocyanin accumulation in *Arabidopsis* and apple (Rubin et al., 2009; Wang et al., 2018). The signaling pathways for anthocyanin induction by nitrogen and phosphorus deficiency are well-characterized for *Arabidopsis*, with R2R3MYBs being the key activating transcription factors (Lillo et al., 2008; Rubin et al., 2009). Induction of auronidin in Marchantia by nitrogen and phosphorus also requires an R2R3MYB (Kubo et al., 2018), suggesting signaling components may be conserved. For Antarctic liverworts and mosses UVB exposure also induced production of red flavonoids, which most commonly were cell wall-bound (Newsham et al., 2005; Waterman et al., 2018). UVB induces anthocyanin production in some angiosperms, but it is much less common a response than induction of flavones/flavonols. Flavones and flavonols are more effective at screening UVB than anthocyanins, although aromatic acylation can give anthocyanins absorbance maxima in the UV range. The induction of anthocyanins by UVB has thus been suggested to be more for ROS scavenging and/or screening of white light than for UVB screening. In the case of the non-acylated cell wall-bound flavonoid pigments of mosses and liverworts, it seems probable that production is induced to screen white light and prevent further ROS generation, especially as the summer conditions in the Antarctic present a combination of stresses from continuous white light, cold, and drought.

There are other red/purple plant pigments besides the flavonoids able to screen in photosynthetically active wavelengths. Notable among these are the betacyanins, which are produced in the many species of the core Caryophyllales that do not produce anthocyanins (Polturak and Aharoni, 2019), and the phenolic pigments of algae. *Zygogonium ericetorum* is a charophyte green alga that can grow in alpine environments and when exposed to abiotic stress produces vacuolar-localized purple pigments, thought to be polymers of glucose and gallic acid, which can absorb in both UVB and photosynthetically active wavelengths (Aigner et al., 2013). In brown algae, phlorotannins can accumulate to more than 15% of dry weight (Imbs and Zvyagintseva, 2018). Phlorotannins are highly hydrophilic polymers, and may be cell wall-bound, stored intracellularly in vesicles, or exported.

Progress on determining the biological roles of cell wall-bound pigments in bryophytes has been limited by the lack of genetic systems, and the difficulty of extracting the pigments. However, genetic tools are now available in Marchantia that will allow tests of the functions of the pigments in abiotic or biotic stress tolerance. Mutants are available that have loss of auronidin pigmentation but retain flavone production (Albert et al., 2018; Kubo et al., 2018), have loss of flavone production but retain auronidin pigmentation, or have reduced amounts of both compounds. These have been used for physiological studies with respect to UVB tolerance (Clayton et al., 2018) and pathogen attack (Carella et al., 2019). In angiosperms, a range of flavonoids are localized to the cell wall (Agati et al., 2012), including rare examples of cell wall-bound anthocyanins (Philpott et al., 2009), although the physiological roles of these are generally unclear. The cell wall localization of other phenylpropanoids, in particular HCA derivatives, is common in angiosperms. These may contribute to lignin formation or be accumulated as monomers or dimers in the wall. Besides having structural roles these polymers may also contribute to physical barriers to pathogens (Zhao and Dixon, 2014). Although lignin is thought to be absent from non-vascular plants, cinnamic acid derivatives such as rosmarinic acid and (neo)lignans are common in bryophytes (Asakawa et al., 2013; Asakawa, 2017), and may be cell wall-localized (Wang et al., 2013). In *Sphagnum* moss, oxidative derivatives of sphagnum acid, *p*-hydroxyacetophenone, hydroxybutenolide, and *p*-hydroxybenzoic acid, as well as the phenolics *p*-coumaric acid and *trans*-cinnamic acid, were predominantly bound to the cell wall (Verhoeven and Liefveld, 1997). It seems a strong possibility that the red flavonoid pigments of bryophytes contribute, along with the cinnamic acid derivatives, to forming a physical barrier against pathogens. The recent study of Carella et al. (2019) demonstrated that the production of auronidin in Marchantia greatly enhanced resistance to *Phytophthora palmivora* infection, with a lack of hyphae penetration into the highly pigmented regions of plants. In relation to the mechanism of action, it would be of much interest to determine the nature of the incorporation of auronidin and sphagnorubins into the wall and whether polymerization occurs. Dimers of auronidin/riccionidin A have been isolated (termed riccionidin B) (Kunz et al., 1993), providing a basis for polymerization.

**Figure 4:**
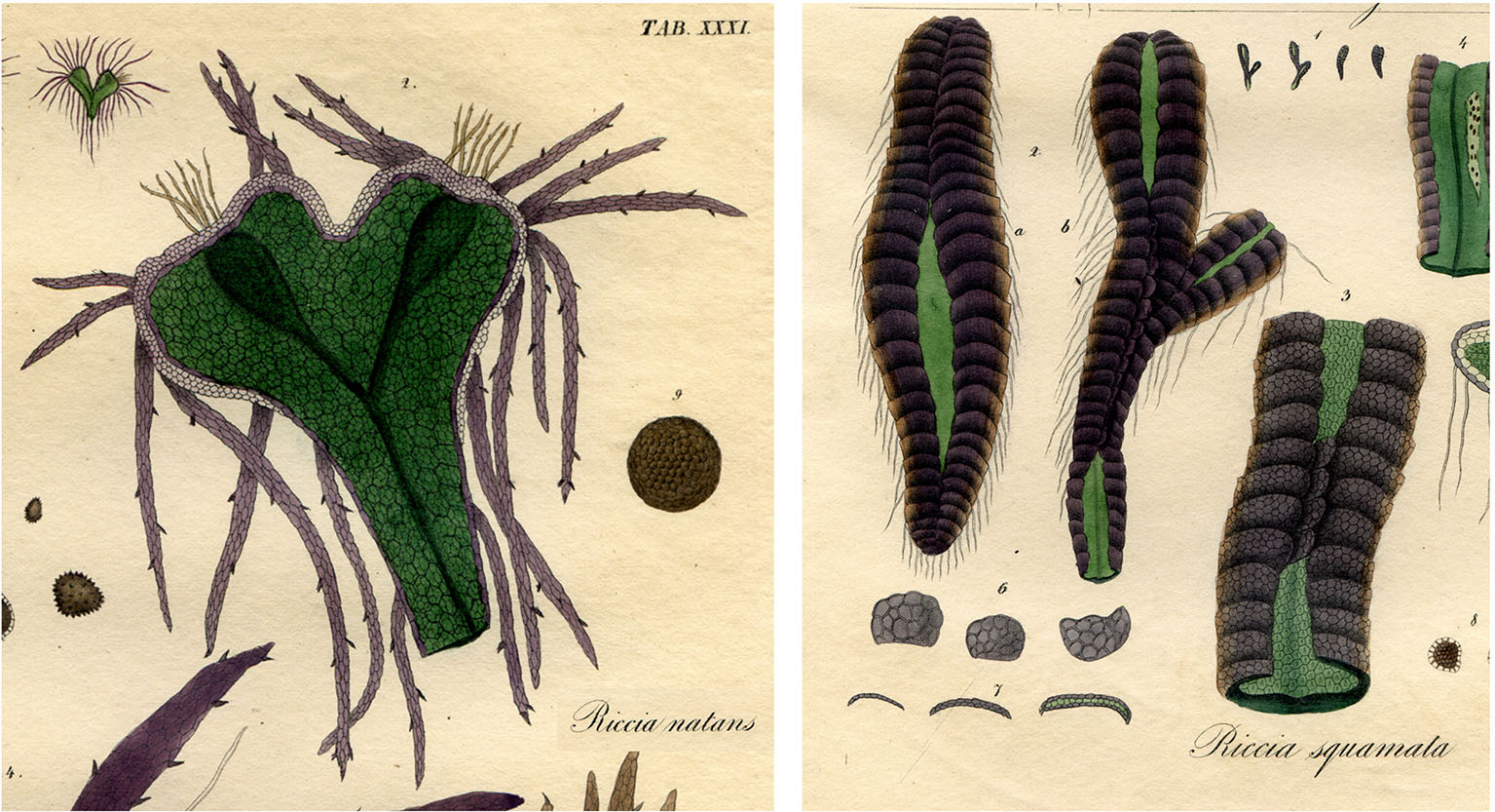
Illustrations of some of the variations in form of the purple-pigmented ventral scales of thalloid liverworts. **Left** The aquatic form of *Ricciocarpos natans* (previously *Riccia natans*) has long ventral scales that extend into the water below the plant. **Right** *Riccia squamata* is one of the drought-adapted thalloid liverworts. As the environment dries out it curls over so that the ventral scales encase the thallus. Illustrations are from Lindenberg (1836).

Several thalloid liverwort genera, and many moss genera, have species with considerable drought tolerance, with examples in both plant groups of individuals withstanding continuous desiccation for more than 20 years (Breuil-Sée, 1993; Stark et al., 2017). As the plants of liverwort genera such as *Riccia* and *Targionia* dry out, the sides of the thallus roll over the dorsal surface so that it is covered by the darkly pigmented ventral scales and rhizoids [see **Figure 4** and Reeb et al. (2018) for examples]. This forms a “capsule” that can recover and renew growth even after extended periods without additional water. The function of the very strong pigmentation of the ventral scales, presumably by cell wall-bound auronidin, is not known. It may provide protection of the DNA against UVB damage during a period when DNA repair mechanisms are not active, given that auronidin accumulation is induced by UVB in some Antarctic species. Alternatively, the modification of the cell wall could prevent pathogen ingress, as demonstrated for Marchantia (Carella et al., 2019), or reduce water loss. Plants of desiccation-tolerant species in leafy liverwort genera such as *Herbertus* and *Cephaloziella* also often have dark red pigmentation (Vitt et al., 2012). A related but little studied example first described in the 1890s (Campbell, 1896) is the formation of “tubers” by some liverwort species, notably *Geothallus tuberosus*. *G. tuberosus* can form thickened inner regions of the thallus that are presumed to store carbohydrates. As the tubers form, the associated cells become strongly dark red pigmented with “thick walls”. The tubers can become buried in the soil and, although the surrounding plant may die, the thallus and associated meristem survive the long dry season of the Southern Californian regions to which the species is native.

**Figure 5:**
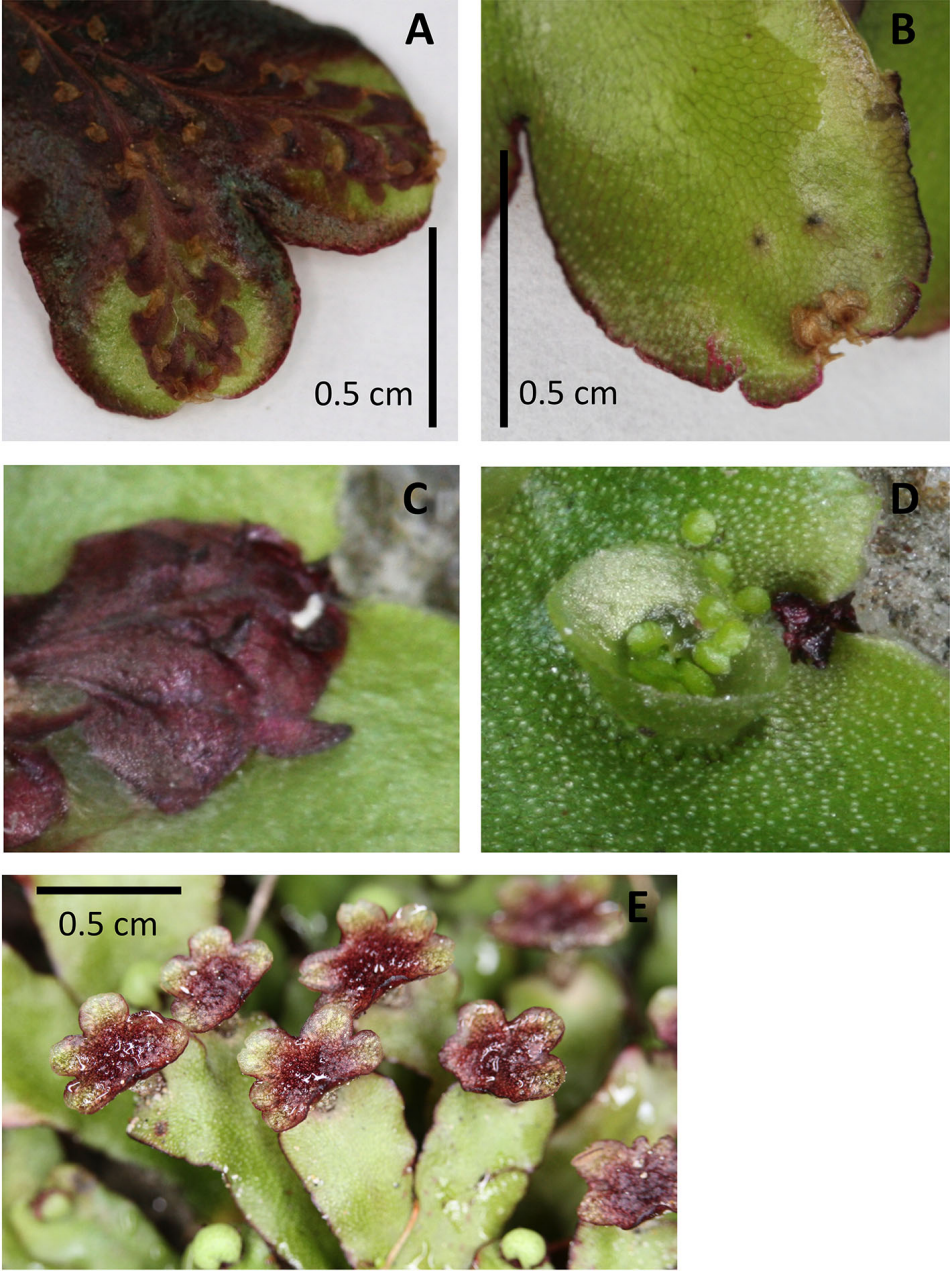
Examples of pigmentation of the thallus and antheridiophores of *Marchantia foliacea*. Ventral **(A, C)** and dorsal **(B, D)** views of a thallus branch. The ventral surface contains the strongly pigmented scales. These extend past the meristematic notch and can be seen from the dorsal surface. The thallus in image **(A)** shows the strong pigmentation that can also occur in the non-scale cells. Image **(D)** shows a close up of the meristem region from the dorsal side, with the corresponding ventral view **(C)** “flipped” vertically to present the same orientation. Image **(E)** shows the strong pigmentation common for the surface of the antheridiophores.

The ventral scales of many thalloid liverworts, frequently strongly pigmented by auronidin, often extend around the apex of the thallus to provide a barrier layer between the meristem and the soil (**Figure 5**). Protection of the meristem from physical damage and pathogen ingress could explain this pigmentation. This suggestion could be extended to include protection against herbivory. The extended ventral scales of the aquatic form of *R. natans* also have strong auronidin-based purple pigmentation (**Figure 4**), so perhaps auronidins contribute to aquatic herbivore deterrence in these cells.

There are few studies on the biological functions of the 3-deoxyanthocyanins that are common in mosses and ferns, but there is evidence they also are involved in plant defense. Greater amounts of 3-deoxyanthocyanins in fronds of the aquatic fern *Azolla* correlated with increased feeding deterrence to snails and the tadpoles of frogs (Cohen et al., 2002a). In the same species, 3-deoxyanthocyanins also may promote the establishment of the symbiosis with the cyanobacterium *Nostoc* (Cohen et al., 2002b). This suggests there are specific biological functions of the flavonoid pigments in different bryophyte and fern species, although the induction of 3-deoxyanthocyanin production in ferns by general abiotic stresses also indicates a general light screening/antioxidant function in common with that of 3-hydroxyanthocyanins in angiosperms. Perhaps the cell wall-bound pigments of the bryophytes have evolved to have elegant multi-functionality, providing abiotic stress tolerance through antioxidant and/or light screening actions and altering physical properties of the cell for biotic stress resistance.

### The Evolutionary Significance of the Occurrence of Different Flavonoid Structural Groups

The identification of different flavonoid groups across land plants has been conducted for many years, both to further understand the evolutionary significance of flavonoid distribution by chemotaxonomy and for the discovery of novel bioactives (Markham, 1988; Asakawa, 2017; Jiang et al., 2016; de Vries et al., 2017; Yonekura-Sakakibara et al., 2019). Flavones and/or flavonols are almost ubiquitous across land plants (Berim, 2016) but variations in the specific types of flavonols or flavones produced have occurred during evolution, for example resulting in the rarity of flavone *O*-glycosides in leafy liverworts (Markham, 1988) or of polymethoxylated flavones in gymnosperms (Berim, 2016). Overall, it is probable that their biosynthesis was acquired very early during land plant evolution as an important stress adaptation. The remarkable exception is the hornworts. Hornworts produce polyphenolics, notably rosmarinic acid and lignan-like compounds (e.g., anthocerotonic acid and megacerotonic acid) (Petersen and Simmonds, 2003; Soriano et al., 2018b) (**Figure 1**), but there is no report of any flavonoid being found (Markham, 1988). Thus, either hornworts diverged from the last common land plant ancestor before the evolution of the flavonoid pathway, or the ability to make flavonoids was subsequently lost in this lineage. The completion of a hornwort genome sequence (Szövényi et al., 2015) and transcriptomic studies examining land plant evolution (Wickett et al., 2014; Puttick et al., 2018) may provide the data to help in resolving this question. Analysis of the transcriptomic (SRA PRJEB21674) and genomic (SRA ERR771108 and SRR1278954 for *Anthoceros agrestis* and *Anthoceros punctatus*, respectively) data currently available on GenBank can identify with confidence hornwort deduced sequences corresponding to the early steps of the phenylpropanoid and flavonoid pathway, including for PHENYLALANINE AMMONIA LYASE (PAL), CINNAMATE 4-HYDROXYLASE (C4H), 4-COUMARATE-COA LIGASE (4CL), CHS, and CHALCONE ISOMERASE-LIKE (CHIL) (**Supplementary Table 1**). However, additional analysis is required to show whether these produce functional enzymes. No clear CHALCONE ISOMERASE (CHI)-encoding sequence is present in the data, but mosses can make flavonoids without a gene corresponding to the typical *CHI* (Cheng et al., 2018). Rosmarinic acid is also found in some algae (Agregán et al., 2017), but comparison of the biosynthetic pathways between land plants and algae has not been made.

Until the recent clarification of the riccionidin structures as auronidins, anthocyanins were thought to be present in all extant lineages of land plants except hornworts. A progression in anthocyanin complexity was suggested, with liverworts producing “primitive” anthocyanidins (the non-glycosylated anthocyanin core), mosses and ferns 3-deoxyanthocyanins, seed plants the 3-hydroxyanthocyanins, and angiosperms a great range of substituted anthocyanins (including 5'-hydroxylation and variation in glycosylation, acylation, and methylation). However, as mentioned earlier, it is now more difficult to speculate on the possible red pigments present in the last common ancestor of land plants. Riccionidin A has been reported from the root cultures of the angiosperm *Rhus chinensis* (syn. *Rhus javanica*) (Taniguchi et al., 2000), but it has not been examined whether this is synthesized *via* an aurone intermediate route.

In addition to the core flavonoid pathway found across most land plants, there are groups of flavonoids prevalent in specific taxonomic groups, such as the isoflavonoids typical of legumes. There are also flavonoid types that occur sporadically, such as aurones that are found in liverworts and some angiosperms. For aurones this may well represent convergent evolution, as even within angiosperms there are alternative biosynthetic mechanisms (Boucherle et al., 2017). New metabolomic technologies combined with additional genome sequences for non-angiosperm species should help clarify the distribution across the land plants of different flavonoid types and the associated biosynthetic genes (Yonekura-Sakakibara et al., 2019).

### The Phenylpropanoid Biosynthetic Pathway in Bryophytes

The core steps of the phenylpropanoid pathway through to the first flavonoids (the chalcones) are conserved across land plants (Tohge et al., 2013), including the presence of *PAL*, *C4H*, *4CL*, *CHS,* and *CHIL* gene sequences in hornworts. Sequences relating to some of these genes are present in the genome sequences of charophyte and chlorophyte algae (Labeeuw et al., 2015; de Vries et al., 2017), but without functional assays the conclusions that can be drawn are limited. Most phenylpropanoid pathway enzymes are thought to have evolved from primary metabolism enzymes (Tohge et al., 2013; Yonekura-Sakakibara et al., 2019), and so related sequences might be expected to be present. For *PAL*, whether it arose during land plant evolution or is an ancestral gene from algae has yet to be resolved (de Vries et al., 2017). It was suggested that *PAL* was acquired by the land plant ancestor *via* a horizontal gene transfer event (Emiliani et al., 2009), but genes related to *PAL* are present in the charophyte *Klebsormidium flaccidum* and could have been acquired by endosymbiotic gene transfer from cyanobacteria to algal ancestors of land plants (de Vries et al., 2017). C4H, which belongs to the CYP73A sub-family of cytochrome P450 monooxygenases (Cyp450s), shows strong sequence conservation across land plants, including characteristic motifs and residues, but no authentic gene sequences are apparent in chlorophyte genomes (Tohge et al., 2013; Davies et al., 2020). In contrast, sequences with similarity to *4CL* do occur in rhodophyte and chlorophyte genomes (Labeeuw et al., 2015; de Vries et al., 2017), suggesting the existence of this enzyme in a shared ancestor of land plants and algae before the ancestral divergence of the red algae (Labeeuw et al., 2015). A further aspect yet to be addressed is the presence in fungi of genes with significant sequence similarity to those of the phenylpropanoid pathway (Bilska et al., 2018; Lu et al., 2019). As the separation of fungi and plants is thought to have occurred during the early stages of eukaryote divergence (Burki, 2014; Burki et al., 2020), it is possible that these may represent cases of convergent evolution.

The type III PKS superfamily that contains *CHS* is present in all plant genomes examined to date (Pandith et al., 2020). PKS genes are found also in fungi, and some bacteria and algae, and the plant PKS genes contain conserved structural elements with the bacterial PKS genes involved in primary metabolism. Across plants there is a wide variety of PKS enzymes with close sequence similarity to CHS but which either use alternative substrates (such as acridone synthases and pyrone synthases) or catalyze different cyclisation reactions using the same starter molecules (notably, STILBENE SYNTHASE, STS). It is thought that STS has independently evolved from CHS several times in the course of evolution (Yonekura-Sakakibara et al., 2019; Pandith et al., 2020). It is probable that there are many novel PKS activities still to be discovered in plants, including bryophytes. This may include steps in bibenzyl biosynthesis, a group of liverwort phenylpropanoid compounds related to plant defense that includes cannabinoid-like structures (Hussain et al., 2018). The presence of at least 24 PKS genes in the Marchantia genome suggests potential biosynthetic diversity, and at least one gene (*Mapoly0014s0122*) is closely related to the anther-specific chalcone synthase-like enzymes (ASCLs) involved in the biosynthesis of sporopollenin in angiosperms (Bowman et al., 2017). However, the majority of the annotated Mp*PKS* genes appear to have resulted from a strongly conserved duplication of a *CHS/PAL* gene pair (Bowman et al., 2017).

The occurrence and function of CHI and CHIL in basal plants is proving to be an interesting question. Liverworts have both types of gene, and knockout *chi* mutants of Marchantia completely lose production of flavones (Clayton et al., 2018). Thus, in Marchantia, as in angiosperms examined, CHI is an essential *in planta* activity for flavanone production. However, no gene sequences for CHI have been found in moss or hornwort genome sequences or transcriptomes (Ngaki et al., 2012; Cheng et al., 2018; Berland et al., 2019). Although spontaneous closure to form the C-ring to produce flavanones from chalcones has been shown to occur *in vitro*, comparative studies on the spontaneous and enzyme catalyzed reactions suggest this is unlikely to be significant *in planta* (Jez and Noel, 2002). Studies on mutants for *chi* in *Arabidopsis* (*tt5*), carnation (*i*), and rice (*gh1*) found that flavonoid biosynthesis was not fully prevented (Stich et al., 1992; Hong et al., 2012; Jiang et al., 2015), suggesting some spontaneous conversion. However, in the case of carnation at least, the residual production of flavanones in the *chi* mutant has been found to be due to a second, weakly expressed, *CHI* gene (Miyahara et al., 2018). Thus, how flavonoid biosynthesis occurs in mosses is an open question. CHI and CHIL are thought to be examples of the rare event of catalytic activity arising in a noncatalytic scaffold protein (Kaltenbach et al., 2018). The mechanism of action of CHIL is unclear, and it may have differing activities across land plants, perhaps based on the promotion of activity of different biosynthetic enzymes through protein-protein interaction. In hop (*Humulus lupulus*), HlCHIL2 enhances the activities of CHS and an aromatic prenyltransferase (HlPT1L) through protein–protein interaction (Ban et al., 2018), and the promotion of flavonol and proanthocyanidin biosynthesis in *Arabidopsis* is proposed to be through direct interaction of CHIL and CHI (Jiang et al., 2015). In Marchantia, CHIL may interact with CHS or more than one phenylpropanoid pathway enzyme, since the production of both flavones and auronidins in *chil* mutants is only about 10% of wild-type amounts (Clayton et al., 2018). Thus, one possibility is that in mosses and hornworts CHIL can replace CHI. However, the moss CHIL genes assayed to date do not have CHI activity (Cheng et al., 2018), making this less probable.

Two major hydroxylase groups, the Cyp450s and 2-oxoglutarate dioxygenases (2OGDs, divided into the three classes DOXA, B, and C), contribute several enzymes to the phenylpropanoid pathway of angiosperms. Cyp450s include C4H, FLAVONOID 3'-HYDROXYLASE (F3’H), and FLAVONE SYNTHASE II (FNSII). 2OGDs include the FLAVANONE 2-HYDROXYLASE (F2H), FLAVANONE 3-HYDROXYLASE (F3H), FLAVONOL SYNTHASE (FLS), FLAVONE SYNTHASE I (FNSI), and ANTHOCYANIDIN SYNTHASE/LEUCOANTHOCYANIDIN DIOXYGENASE (ANS). The evolutionary aspects of these gene families with regard to flavonoid biosynthesis were recently reviewed by Yonekura-Sakakibara et al. (2019). C4H is conserved in bryophytes, and the presence of all the other enzymes in liverworts and/or mosses would be expected based on the compounds produced. However, the close similarity of the sequences within the Cyp450 and 2OGD enzyme groups means that assignments based only on sequence similarity to the angiosperm genes should be treated with caution, and conclusive identification of other genes requires functional analysis. FNSI, F2H, and F3H have high sequence similarity and are in the DOXC28 clade and FLS and ANS are close in sequence and in the DOXC47 clade. A review of the two clades, and possible evolutionary timing of the origin of each, is given in Yonekura-Sakakibara et al. (2019).

Based on the occurrence of flavones in liverworts and mosses, it is expected that F3H and FNS activities evolved early in land plants, and two DOXC28 genes have increased transcript abundance during UVB-induced flavone production in Marchantia (Clayton et al., 2018). However, to date, the only functional characterization is for a F2H that may contribute to flavone biosynthesis in the liverwort *Plagiochasma appendiculatum* (Han et al., 2014). The biosynthesis of flavones illustrates the difficulties of making assumptions about gene function, as a variety of alternative routes to flavone *O*- and *C*-glycosides have evolved in angiosperms (Jiang et al., 2016). A further complication is that the 2OGD enzymes (particularly the FLS and ANS) show promiscuous and sometimes overlapping activities when assayed *in vitro* (reviewed in Martens et al., 2010). Studies with *Arabidopsis* have shown that these “secondary” activities can also be present *in planta*, as ANS can contribute to (relatively weak) flavonol biosynthesis in the *Arabidopsis fls-1* mutant (Martens et al., 2010).

As yet, it is not clear precisely what phenylpropanoid biosynthetic activities may be present in bryophytes but not found in other plant groups. There are certainly some major pathway branches prevalent in bryophytes that are absent or rare in other groups, such as those for bibenzyls, auronidins, and sphagnorubins. Corresponding evolutionary divergence of specialized metabolic pathways would be expected to underpin the occurrence of the differing compound types. Phylogenetic analysis of the 148 Cyp450, 38 2OGD, and 41 Family-1 UDP-glycosyltransferase (the UGT family containing the “plant secondary product glycosyltransferase” motif) genes of Marchantia found that the majority formed individual clades that also suggested substantial lineage-specific diversification of specialized metabolism (Bowman et al., 2017). Moreover, the emerging transcriptome and genome sequence information from bryophytes is suggesting expanded functionality may have occurred for other classes of enzymes involved in phenylpropanoid biosynthesis. In the next section we examine two specialized metabolism gene families that show unexpectedly large gene family sizes in the Marchantia genome: those for POLYPHENOL OXIDASE (PPO) and DIRIGENT (DIR) proteins.

### Liverworts May Have Expanded Functional Roles in Specialized Metabolism for Polyphenol Oxidase and Dirigent Proteins

**Figure 6:**
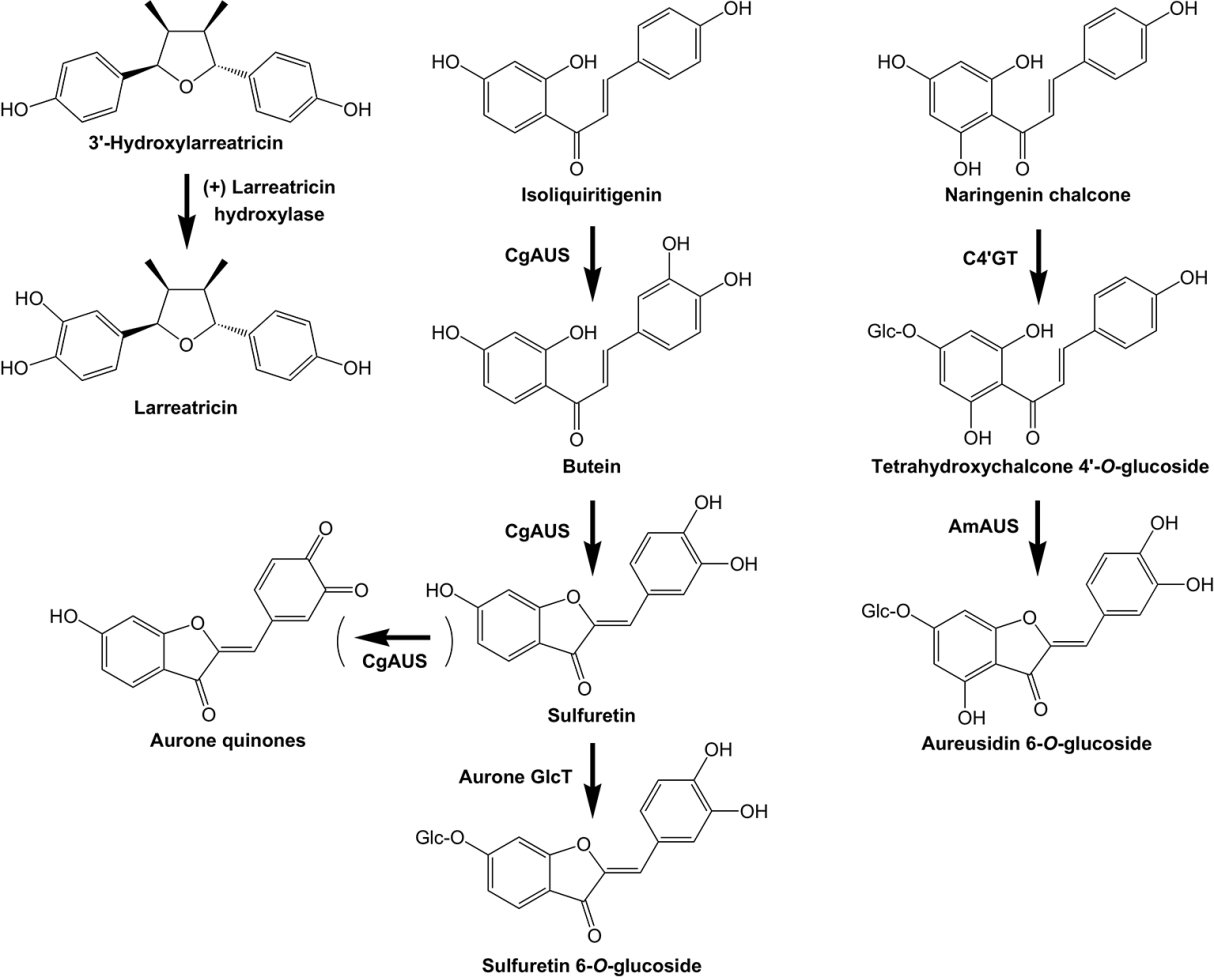
Examples of plant POLYPHENOL OXIDASES with specific activities in specialized metabolism. The biosynthetic activities of three PPOs are shown: (+) Larreatricin hydroxylase, which is part of lignan biosynthesis in the creosote bush (*Larrea tridentata*), and the AURONE SYNTHASE from *Coreopsis grandiflora* (*Cg*AUS) and *Antirrhinum majus* (*Am*AUS).

PPO genes are found throughout land plants, as well as in bacteria, fungi, and animals, but are absent from algae. PPOs are type-III-copper proteins and the name PPO covers two major enzyme types: tyrosinases, which hydroxylate *para*-substituted monophenols to *ortho*-diphenols (monophenolase activity) and use molecular oxygen to oxidize *ortho*-diphenols to *ortho*-quinones (diphenolase activity); and the catechol oxidases, which have only the diphenolase activity. However, it has been recently proposed that monophenolase activity could be a widespread feature of PPOs, but that the activity has remained cryptic because activity assays usually use tyrosine rather than the natural substrates, which are often not known (Molitor et al., 2016). PPOs are commonly thought of as plant defense enzymes that oxidize and/or polymerize a range of phenolic substrates with which they come into contact during cell disruption, resulting in the familiar browning reactions following tissue damage, for example in cut apples or potatoes. However, in addition to these general activities, some PPOs can conduct cross-linking reactions in biosynthetic pathways, such as latex formation; and new specific roles for PPOs have emerged in recent years (**Figure 6**). The published PPO gene family size in plants varies from zero (e.g., *Arabidopsis*) to 13 in *Physcomitrella patens* (Tran et al., 2012). Several angiosperm species examined have only a single PPO gene, but 11 genes have been found in genome sequences of *Glycine max* (the legume soybean), *Populus trichocarpa* (poplar), and *Selaginella moellendorffii* (a lycophyte) (Tran et al., 2012). However, we found a much larger PPO gene family in the Marchantia genome: there are 64 candidate PPO genes (including gene fragments and unresolved gene models). Excluding those having partial gene models, 46 of the 64 PPO genes were represented in the RNA-seq data of Berland et al. (2019) and so are actively transcribed. Given the relatively small number of total gene models in the draft genome sequence of Marchantia, this represents a significant gene family, larger than the annotated 2OGD and UGT families.

**Figure 7:**
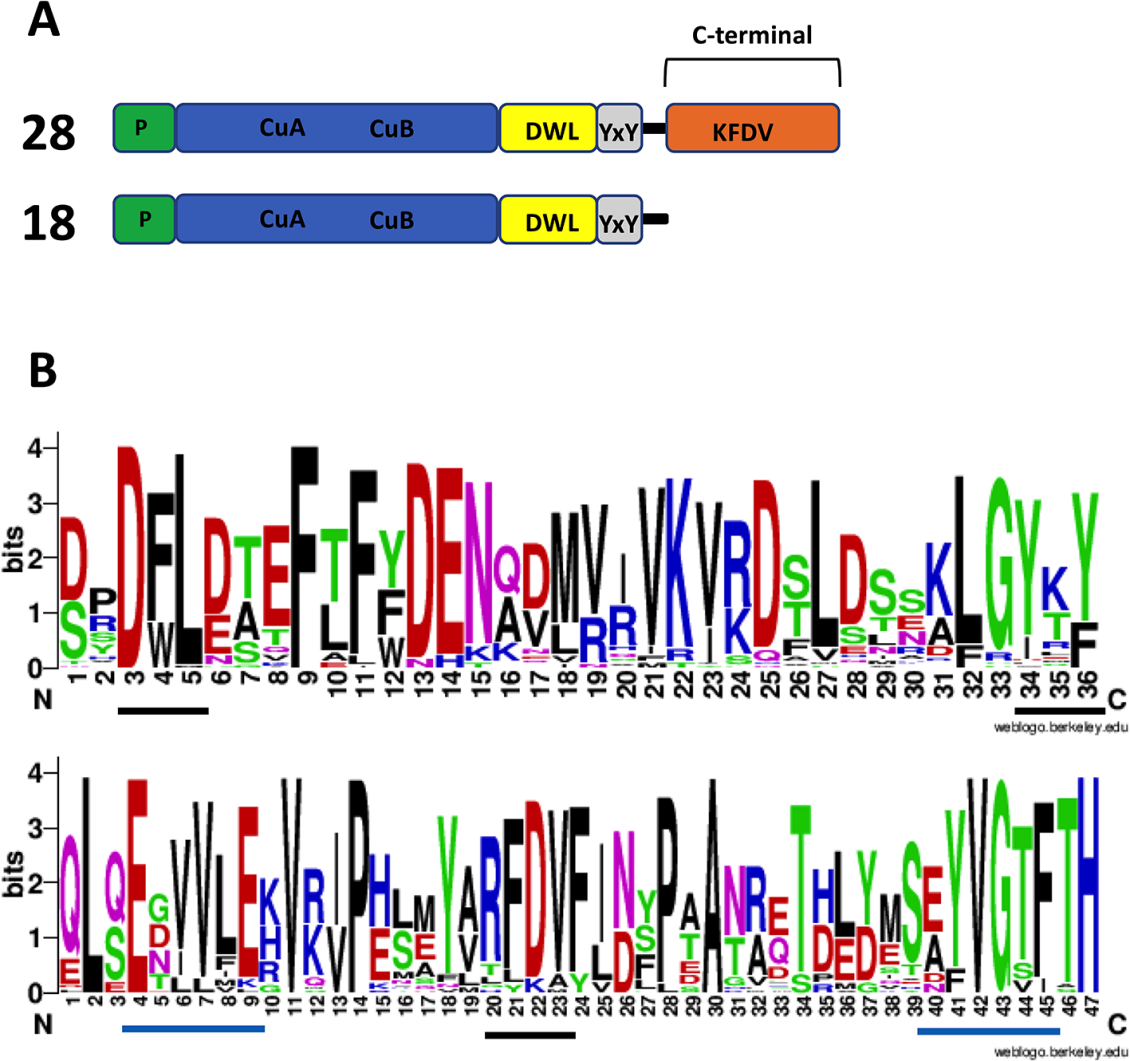
Amino acid sequence features of polyphenol oxidases (PPOs) in *Marchantia polymorpha*. **(A)** Number of expressed PPO gene models found and their PPO type. RNA-seq data from Berland et al. (2019) were used to check for expression. The top structure (not to scale) is standard for plant PPOs. Transit/signal peptide (P), copper binding domain TYR : PFAM 00264 (CuA/CuB), PPO1_DWL: PFAM 12142 domain (DWL), tyrosine motif (YxY), PPO1_KFDV : PFAM12143 domain (KFDV). **(B)** Weblogo display of conserved residues of DWL and KFDV domains based on all gene models having an intact region (generated using https://weblogo.berkeley.edu/logo.cgi). DWL, tyrosine, and KFDV core motifs are underlined in black. Residues for the regions identified by Tran et al. (2012) with core motifs of EEEVLV (left) and EFAGSF (right) are underlined in blue.

Plant PPOs characterized to date are produced in a latent state as proteins of about 64–68 kDa. Besides the N-terminal targeting peptide (usually for plastid localization), PPOs contain a catalytically active domain of about 40 kDa, and a C-terminal domain of about 19 kDa that shields the active site and is later cleaved off to release the active protein. The C-terminal domain is ubiquitous in plant PPOs examined to date. Based on predicted amino acid sequences, PPOs with this typical structure are found in Marchantia; however, there are also members of the PPO family that lack this C-terminal domain (**Figure 7**), including the auronidin-related *Mapoly0021s0041*. These “short” type PPOs have also been found in fungi and bacteria (Huber et al., 1985; Shuster and Fishman, 2009; Gasparetti et al., 2010). Only a few of this short type, from the bacteria *Streptomyces* and *Bacillus*, have been extensively studied. The *Streptomyces* PPO is thought to be initially in an inactive form that is bound with a “caddie” protein. The caddie protein subsequently transfers copper to the PPO and disassociates to release an active PPO (Chen et al., 1992; Matoba et al., 2006). In contrast, the PPO from *Bacillus* does not need a caddie protein (Sendovski et al., 2011).

The first PPO found to have an unexpected role in plant specialized metabolism was the AUREUSIDIN SYNTHASE (AUS) that converts chalcone 4'-*O*-glucosides to aurone 6-*O*-glucosides in *Antirrhinum majus* (Nakayama et al., 2000; Davies et al., 2006; Ono et al., 2006; Elumalai and Liu, 2011). AmAUS differed from previously characterized PPOs in three important aspects: it was vacuole localized (Ono et al., 2006), it was a glycoprotein, and it lacked activity against common PPO substrates such as tyrosine or 3,4-dihydroxyl L-phenylalanine (L-DOPA). AmAUS conducts oxygenation of the B-ring of the chalcone, which is followed by cyclization into the aurone (Nakayama et al., 2000) (**Figure 6**). Although it can use chalcone aglycones *in vitro*, *in planta* aurone production in *A. majus* requires the activity of the CHALCONE 4'-*O*-GLUCOSYLTRANSFERASE (C4’GT) since only the glucoside is transported into the vacuole (Ono et al., 2006; Bradley et al., 2017). Subsequently, PPOs that form aurones were identified in other species, with the AURONE SYNTHASE of *Coreopsis grandiflora* that makes 4-deoxyaurones being studied in detail (Molitor et al., 2015; Molitor et al., 2016). In contrast to AmAUS, the CgAUS has the N-terminal chloroplast transit peptide and thylakoid transfer domain characteristic of plastid-localized PPOs involved in browning reactions, and uses chalcone aglycones to make aurone aglycones (Kaintz et al., 2014; Molitor et al., 2015). A PPO (*Mapoly0021s0041*) is strongly up-regulated by MpMYB14 in association with auronidin production in Marchantia (Berland et al., 2019). Loss-of-function *mapoly0021s0041* mutants have greatly reduced amounts of auronidin, suggesting it too may encode an aurone biosynthetic activity, or is involved in later steps of auronidin biosynthesis and/or polymerization. Aurones have been found across land plant groups, but with sporadic occurrence. This suggests that their biosynthesis may have arisen independently on a number of occasions. Although both the aurone biosynthetic enzymes characterized to date are PPOs, the sequences are phylogenetically distinct and have differing activities and sub-cellular localization. Additionally, the biosynthesis of the aurone hispidol in *Medicago truncatula* may be conducted by a peroxidase rather than a PPO (Farag et al., 2009). Besides AUS, PPOs have been implicated in tyrosine or phenylpropanoid biosynthetic pathways of walnut (Araji et al., 2014) and creosote bush (*Larrea tridentata*) (Cho et al., 2003).

Forty of the Marchantia PPO genes (66%) occur as tandem repeats or small gene clusters (local tandemly arrayed genes; TAGs). Although this figure may be either an under- or over-estimate as it is based on the initial scaffold assembly of the genome (Bowman et al., 2017), it is nevertheless a much higher value than the overall percentage of Marchantia TAGs estimated on the same basis, which at 5.9% is near the lower end of the range observed in flowering plants (Bowman et al., 2017). The TAG percentage is also relatively high for some of the other characterized specialized metabolite gene families of Marchantia. For example, there are 18 occurrences of neighboring PAL and/or CHS genes. TAGs are notable in some angiosperm species that have prominent specialized metabolic characteristics—such as the terpenoid pathways of the tree species *Eucalyptus grandis* and teak (*Tectona grandis*). Teak has at least 14 TAGs for the terpene synthase gene family (Zhao et al., 2019). *E. grandis* has the largest number of genes in tandem repeats reported among sequenced plant genomes, at 34% of total genes (Myburg et al., 2014). For the Marchantia phenylpropanoid biosynthetic pathway, 10 multigene families have expanded, mostly through tandem duplication, to result in a total of 174 genes. In angiosperms, gene diversification is a result of a combination of local duplication events and whole genome duplications, but it is probable that no whole-genome duplication events have occurred during liverwort evolution (Bowman et al., 2017). Therefore, although there is no overall increase in the frequency of TAGs in liverworts (at least for Marchantia), local gene duplication events are likely to have been a common mechanism for generating gene neofunctionalization in specialized metabolism. Whether this is typical of other liverworts requires the completion of further genome sequences. However, BLAST analysis of the *Lunularia cruciata* transcriptome (www.polebio.lrsv.ups-tlse.fr/Luc_v1/Luc_v1.fa) identified more than 20 sequences with the conserved features of PPOs (data not shown), suggesting a large gene family in this species also.

Dirigent proteins (DIR) are small (~16–18 kDa) cell wall-localized proteins that may control the regio- and stereospecific outcome of phenoxy radical coupling in lignin and lignan polymerization reactions (Davin et al., 1997; Gang et al., 1999). The polymerization reactions also require the activity of laccase or peroxidase to produce electron oxidative capacity to generate the phenoxy radical. In vascular plants, lignins are complex, amorphous heteropolymers involved in wall strengthening and pathogen resistance, with species-specific composition produced by polymerization of coniferyl, sinapyl, and *p*-coumaryl alcohols. A role for DIRs in directing the reactions leading to the formation of lignin has been proposed but not definitively established, although there is strong genetic evidence in support of some specific cases (Hosmani et al., 2013).

The role of DIRs in determining stereospecificity has been best described in the formation of lignans, a class of 8-8’ linked C_6_C_3_ phenylpropanoid dimers involved in pathogen resistance, for example in the production of (+)- or (−)-pinoresinol compounds in flax (*Linum usitatissimum*) and pea (*Pisum sativum*) (Kim et al., 2015; Corbin et al., 2018). The X-ray crystal structure of PsDRR206 involved in (+)-pinoresinol formation suggested that the active protein had a trimeric structure (Kim et al., 2015). Recent work has suggested that at least some DIRs may do more than the hypothesized positioning of phenoxy radicals prior to coupling, and may themselves possess enzymatic activity. The crystal structure of *Arabidopsis* AtDIR6 identified potentially catalytic residues including aspartic acids that were essential for activity, and it was proposed that this protein catalyzed the cyclization of the *bis*-quinone methide intermediate during (+)- or (–)-pinoresinol formation (Gasper et al., 2016). Also, a recombinantly expressed DIR from *Glycyrrhiza echinata* was found to possess isoflavanol dehydratase activity and carry out the final ring-closure step of the biosynthesis of the anti-microbial phytoalexin (–)-pterocarpan (Uchida et al., 2017).

DIR gene families can be quite large, with 26 genes in *Arabidopsis* (Paniagua et al., 2017) and 44 genes in flax (of which seven appeared to be gene fragments or result from chromosomal rearrangements; Corbin et al., 2018). Of the 37 genes with classical DIR structure in flax, 15 paralogous gene pairs were identified. Kubo et al. (2018) identified 52 dirigent-like predicted proteins in the Marchantia genome sequence. Our analysis for this article found that at least 35 of these occur as TAGs. However, the deduced protein sequences of the family members are diverse, and the functionality of the proteins has not yet been established. Our BLAST analysis of the Marchantia genome and transcript resources with the 24 annotated *Arabidopsis* DIR genes gave us 60 initial candidate gene models, with strong evidence of some very recent gene duplications giving groups of adjacent genes with highly similar or identical deduced amino acid sequences.

Phenylpropanoid biosynthesis and lignification are common plant responses to biotic and abiotic stress (Zhao and Dixon, 2014; Paniagua et al., 2017) and consequently, as a component of lignification, DIRs have been implicated in responses to pathogen and drought stress (e.g., Thamil Arasan et al., 2013; Paniagua et al., 2017). In *P. patens*, fungal infection resulted in increased incorporation of phenolic compounds into the wall and up-regulation of a *DIR* gene (Reboledo et al., 2015). In Marchantia, abiotic stresses such as UVB irradiation, N deficiency and salinity (Albert et al., 2018; Kubo et al., 2018), and pathogen attack (Carella et al., 2019) increased the expression of Mp*MYB14*. MpMYB14 promotes auronidin production and up-regulates transcript abundance for at least three DIR genes (*Mapoly0006s0216*, *Mapoly0006s0217*, *Mapoly0078s0058*) (Albert et al., 2018; Kubo et al., 2018). The deduced protein products of these genes possess predicted signal peptides (SignalP 5.0, http://www.cbs.dtu.dk/services/SignalP/), indicative of secretion to the vacuole or, extracellularly, to the cell wall. However, prediction of subcellular localization using WoLFPSORT (https://wolfpsort.hgc.jp) indicated with low confidence different compartments for the three predicted protein products of the genes: extracellular (*Mapoly0006s0217*), vacuolar (*Mapoly0006s0216*), and cytoplasmic (*Mapoly0078s0058*). Intracellular coupling of monolignol radicals has been described in *Arabidopsis* (Dima et al., 2015). There have been no studies on DIR genes in hornworts. However, as lignans are prominent specialized metabolites of hornworts, and DIRs have roles in lignan biosynthesis in angiosperms, this could be a worthwhile area to investigate.

### Evolution of the Transcriptional Regulation of the Phenylpropanoid Pathway

In angiosperms and gymnosperms, the key regulatory complex consists of R2R3MYB and bHLH TFs joined with a WD-Repeat (WDR) protein, a composition of proteins known as an “MYB-bHLH-WD repeat (MBW)” complex. The MBW complex that activates anthocyanin and proanthocyanidin production contains R2R3MYB proteins from sub-group (SG) 5 or 6, and commonly promotes transcription of the biosynthetic genes throughout the pathway. The action of the MBW complex is modified by a WRKY class activator TF and a series of proteins with repressor actions (Lloyd et al., 2017). In particular, R2R3MYBs from SG4 can join an activating MBW complex and turn it into one that represses target gene transcription, and R3MYBs can bind the bHLH to prevent it from forming the MBW complex, thus competitively inhibiting activation (Albert et al., 2014). The SG4 R2R3MYBs are characterized by the presence of an ethylene response factor (ERF)-associated amphiphilic repression (EAR) motif (LxLxL or DLNxxP) or a TLLLFR motif in the C terminus that mediates transcriptional repression (Chen et al., 2019a; Chen et al., 2019b; Ma and Constabel, 2019). For the activation of the flavonol and flavone branches, a SG7 R2R3MYB acts without being part of the complex. There is also regulation upstream of the flavonoid pathway, as HY5 activates the production of the SG7 R2R3MYB. Additionally, in *Arabidopsis* it has been shown that HY5 directly activates transcription of some flavonoid biosynthetic genes, such as CHS. The conservation of HY5 function in the UVB responses of both bryophytes and angiosperms was mentioned earlier, although its target gene set has yet to be resolved.

The expansion of TF families during evolution has been a driver of diversity in land plants, as a consequence of multicellularity and increased organismal complexity and/or for coping with the increased stress of a sessile land-based lifestyle. The MYB gene family is one of the largest TF families in plants, with *Arabidopsis* having 137 R2R3MYB genes (Feller et al., 2011). This includes one SG5, four SG6, and three SG7 genes in *Arabidopsis* for proanthocyanidin, anthocyanin, and flavonol production, respectively. The presence of small gene families for sub-groups regulating specialized metabolic pathways is common for angiosperms, and has enabled sub-functionalization and diversification of flavonoid temporal and spatial regulation in flowers, seeds, and vegetative tissues. The bHLH and WDR components are less specific in their regulatory targets, and can regulate other characters as well as flavonoid biosynthesis, such as epidermal cell differentiation.

The great majority of information on the transcriptional regulation of specialized metabolite pathways is available from studies on angiosperms, with only a small number of studies on gymnosperm, fern, or bryophyte species. Identifying the genetic components for flavonoid pathway regulation in these other plant groups will help establish a model for how regulation of specialized metabolism may have changed during evolution. For bryophytes, notable questions relating to flavonoid pathway regulation include: are R2R3MYB and bHLH genes the key direct activators? If MYBs are the direct activators, which SGs are present in bryophytes and do small gene families occur for each SG? Does a MBW complex form in bryophytes? Do repressor TFs modify pathway regulation? Characterizing these aspects in species such as *M. polymorpha*, *P. patens*, and the lycophyte model *S. moellendorffii* should indicate which aspects of flavonoid regulation are conserved across land plants, and thus may have been present in the early land plant ancestor, and which aspects may have arisen as part of evolutionary diversification of the different land plant groups.

Compared with angiosperms, the characterized bryophytes and lycophytes have small TF families. There are only 22, 49, and 62 R2R3MYBs in the genomes of *M. polymorpha*, *P. patens*, and *S. moellendorffii*, respectively (Feller et al., 2011; Bowman et al., 2017). For Marchantia genes, a phylogenetic comparison of this gene family shows that *MpMYB02* and *MpMYB14* fall basal to a clade that contains all the phenylpropanoid-related R2R3MYB genes of *Arabidopsis* (SGs 4, 5, 6, 7, 15, and 44) (Bowman et al., 2017). Concluding whether these correspond to descendants of the flavonoid regulatory R2R3MYBs of the ancestral land plant requires further study, although both MpMYB02 and MpMYB14 activate phenylpropanoid biosynthetic genes. MpMYB02 is required for production of bibenzyls (Kubo et al., 2018) while MpMYB14 is essential for auronidin production and promotes the production of flavone *O*-glycosides (Albert et al., 2018; Clayton et al., 2018; Kubo et al., 2018). The profiles of transcripts up-regulated by Mp*MYB02* and Mp*MYB14* include *DIR* genes (Albert et al., 2018; Kubo et al., 2018; Berland et al., 2019). For MpMYB14, this includes the three *DIR* genes discussed earlier as well as other *DIR* genes that have been shown to be direct targets (Kubo et al., 2018). Co-expression analysis in flax found that MYB TFs were up-regulated along with *DIR* genes during secondary wall biosynthesis (Corbin et al., 2018), suggesting that *MYB* proteins could control *DIR* expression in both angiosperms and bryophytes.

MpMYB14 must act redundantly with other uncharacterized TFs for flavone production, as Mp*myb14* mutants still show the induction of flavones in response to UVB (Clayton et al., 2018), nutrient stress, or high-irradiance white light (Albert et al., 2018). Flavone production is reduced in Mp*hy5* mutants, so it is possible that HY5 is a direct activator of flavonoid biosynthetic genes as in *Arabidopsis*, but there may also be HY5-independent activation pathways for flavone production (Kondou et al., 2019). Analysis of changes in transcriptomes in response to UVB treatment does not present any alternative R2R3MYB candidate for flavone regulation (Clayton et al., 2018). Thus, Marchantia may lack the equivalent of the angiosperm SG7 activators of flavonol and flavone biosynthesis.

The Pa*bHLH* gene of the liverwort *P. appendiculatum* is a probable activator of bibenzyl biosynthesis (Wu et al., 2018). Over-expression of Pa*bHLH* in *P. appendiculatum* increased bibenzyl concentration and up-regulated transcript abundance from known phenylpropanoid biosynthetic genes (*PAL*, *4CL*) and candidate bibenzyl biosynthetic genes, whereas RNA interference-induced suppression down-regulated the same genes and reduced bibenzyl accumulation. Phylogenetically, Pa*bHLH* falls within clades containing the flavonoid MBW *bHLH* sequences of angiosperms (within bHLH subgroup IIIf), suggesting it may be homologous to them. In Marchantia, Mp*bHLH12* is the gene with the highest sequence identity to Pa*bHLH* and the flavonoid-related bHLHs of angiosperms, and transcriptomic analysis of Mp*BHLH12* overexpression transgenics suggests it may also be involved in flavonoid regulation (Arai et al., 2019). However, although *R2R3MYB* and *bHLH* genes do regulate flavonoid biosynthesis in liverworts, and there are conserved WDR sequences in the genome (Bowman et al., 2017), there is no answer yet on whether the MBW complex exists in bryophytes. A flavonoid-related MBW complex has been characterized in the gymnosperm Norway spruce (*Picea abies*) (Nemesio-Gorriz et al., 2017), supporting an origin for the MBW complex in the plant lineage prior to the last common ancestor of gymnosperms and angiosperms, around 350–300 MYA. However, although the conserved amino acid motif ([D/E]Lx2[R/K]x3Lx6Lx3R) identified as necessary for R2R3MYB proteins to bind the bHLH partners (Zimmermann et al., 2004) is present in the *S. moellendorffii* sequence SmXP002978781, it is lacking in bryophyte R2R3MYBs studied to date. The closest matches in *P. patens* (PpXP001752936) and Marchantia (MpMYB02 and MpMYB14) lack one and two deduced amino acid residues, respectively.

Whether bryophytes possess MYB genes with a repressive action in phenylpropanoid regulation, either the R2R3MYB active repressors that form part of the MBW complex or the R3MYBs that are thought to “compete” for the bHLH proteins, is also an open question. We were unable to identify (known) repression motifs in any of the Marchantia R2R3MYB sequences. Both *P. patens* and *S. moellendorffii* have R2R3MYB genes with putative EAR motif sequences (LxLxL), but the possible function of these in regulating phenylpropanoid biosynthesis has not been examined. Analysis of the auxin signaling pathway of Marchantia has identified an orthologue of TOPLESS, which in angiosperms interacts with the EAR motif to mediate transcriptional repression (Flores-Sandoval et al., 2015). The Marchantia genome contains an expanded R3MYB gene family (Bowman et al., 2017), but no analysis of these with regard to flavonoid biosynthesis has been published.

In summary, based on the evidence from Marchantia, it seems probable that the ancestral R2R3MYB regulators of phenylpropanoid metabolism were activators acting outside of an MBW complex. R2R3MYB-repressive TFs and the MBW complex probably evolved after the last common ancestor of liverworts and gymnosperms/angiosperms. As the flavone pathway probably evolved prior to anthocyanin biosynthesis, it could be expected that R2R3MYBs most similar to SG7 might be the ancestral type. However, the specific flavone activators of Marchantia have yet to be identified. MpMYB0*2* and MpMYB14 may correspond to the ancestral phenylpropanoid pathway activators, and like SG7 probably act outside the MBW complex, but it is difficult to state which is the most closely related SG because of the extent of sequence divergence, with no conservation of sequence outside the MYB domains themselves. Furthermore, additional data are required from other bryophyte species, as the evolutionary path to Marchantia will have resulted in extensive genetic changes and the loss of characters that were present in the last common ancestor.

## Concluding Comments

The commonality of phenylpropanoid biosynthetic genes between bryophytes and angiosperms, and the conserved functions of flavonoids in assisting in tolerance to stresses such as UVB and pathogen attack, support the proposal that the pathway arose before the last common ancestor of these land plant groups, relatively early during the process of land colonization. The exception to this is the hornworts, which lack flavonoids. Unless the divergence of hornworts occurred before the pathway arose, the hornwort ancestor must have acquired mutations that caused loss of the biosynthetic or regulatory capacity. This may be analogous to the loss of anthocyanin biosynthesis in some lineages of the Caryophyllales, where they are replaced by betalains. As the main red pigments of angiosperms (soluble anthocyanins) and bryophytes (cell wall-bound auronidins and sphagnorubins) differ in structure and cellular properties, it is difficult to suggest what the original common ancestor may have possessed with regard to red pigments. Establishing which components of anthocyanin biosynthesis are present or lacking in bryophytes may help in this regard.

The diversification of both specialized metabolite biosynthesis and the transcription factors that regulate the pathways are thought to be important contributors to the evolution of plants to occupy the varied ecological niches offered on land (Pichersky and Gang, 2000). To date, much of our understanding of the genetic basis of the diversification process has been based on studies of flowering plants. However, the completion of the first genome sequences for a moss (*P. patens*) and liverwort (*M. polymorpha*) has started to reveal the details of the specialized metabolite gene families, such as for phenylpropanoid biosynthesis. Notably, the TF families thought to regulate the phenylpropanoid pathway are much smaller in Marchantia than in flowering plants. However, there are relatively large Marchantia gene families for enzymes that are often involved in specialized metabolism, such as the Cyp450, 2OGD, and UGT families. Moreover, Marchantia has large PPO and DIR gene families compared to angiosperms, suggesting these enzyme groups may make a greater contribution than previously anticipated to phenylpropanoid and other specialized metabolite biosynthesis in the liverworts. Thus, in liverworts some of the gene families involved in the biosynthesis of specialized metabolites appear to have undergone more gene duplication (allowing consequent sub- and neofunctionalization for particular family members) than the TFs that regulate the same pathways. Expansion of the regulatory TF families through duplication and sub/neofunctionalization is seen in the angiosperms, probably reflecting increased organismal complexity.

## Author Contributions

KD, RJ, YZ, NA, DB, BJ, JB and KS reviewed literature, formulated ideas and wrote the manuscript. KD and KS prepared the figures. KD, RJ, YZ, DB and KS conducted bioinformatic analysis.

## Funding

Financial support was provided by the Marsden Fund of New Zealand Grant PAF1701.

## Conflict of Interest

The authors declare that the research was conducted in the absence of any commercial or financial relationships that could be construed as a potential conflict of interest.
